# Cardiovascular disease and glycemic control in type 2 diabetes: now that the dust is settling from large clinical trials

**DOI:** 10.1111/nyas.12044

**Published:** 2013-02-06

**Authors:** Francesco Giorgino, Anna Leonardini, Luigi Laviola

**Affiliations:** Department of Emergency and Organ Transplantation, Section of Internal Medicine, Endocrinology, Andrology and Metabolic Diseases, University of Bari Aldo MoroBari, Italy

**Keywords:** type 2 diabetes, HbA_1c_, macrovascular disease, blood pressure, lipids, hypoglycemia, glucagon-like peptide-1, ADVANCE, ACCORD, VADT

## Abstract

The relationship between glucose control and cardiovascular outcomes in type 2 diabetes has been a matter of controversy over the years. Although epidemiological evidence exists in favor of an adverse role of poor glucose control on cardiovascular events, intervention trials have been less conclusive. The Action to Control Cardiovascular Risk in Diabetes (ACCORD) study, the Action in Diabetes and Vascular Disease (ADVANCE) study, and the Veterans Affairs Diabetes Trial (VADT) have shown no beneficial effect of intensive glucose control on primary cardiovascular endpoints in type 2 diabetes. However, subgroup analysis has provided evidence suggesting that the potential beneficial effect largely depends on patients’ characteristics, including age, diabetes duration, previous glucose control, presence of cardiovascular disease, and risk of hypoglycemia. The benefit of strict glucose control on cardiovascular outcomes and mortality may be indeed hampered by the extent and frequency of hypoglycemic events and could be enhanced if glucose-lowering medications, capable of exerting favorable effects on the cardiovascular system, were used. This review examines the relationship between intensive glucose control and cardiovascular outcomes in type 2 diabetes, addressing the need for individualization of glucose targets and careful consideration of the benefit/risk profile of antidiabetes medications.

## Introduction

Cardiovascular disease (CVD) is the major cause of death in patients with type 2 diabetes (T2D), as more than 60% of T2D patients die of myocardial infarction (MI) or stroke, and an even greater proportion of patients have serious burdensome complications.^1^ The impact of glucose lowering on cardiovascular complications is a hotly debated issue. The United Kingdom Prospective Diabetes Study (UKPDS) was the first clinical trial to provide key evidence of the importance of using intensive therapy for diabetes control in individuals with newly diagnosed T2D. However, although the insulin or sulphonylurea-based intensified glucose-control treatment was effective in reducing the risk of major microvascular endpoints, the effects on CVD risk were modest and did not reach statistical significance.^2^ Recent large clinical trials (often referred to as “megatrials”), the Action in Diabetes and Vascular Disease (ADVANCE),^3^ Action to Control Cardiovascular Risk in Diabetes (ACCORD),^4^ and the Veterans Affairs Diabetes Trial (VADT),^5^ reported no significant decrease in primary cardiovascular endpoints with intensive glucose control.

**Figure 1:**
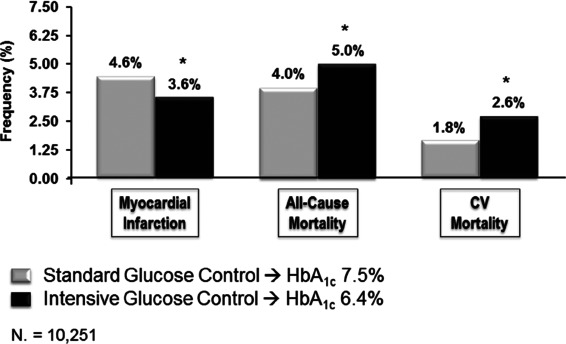
Effects of intensive glucose control on all-cause and cardiovascular mortality and myocardial infarction in the ACCORD study. CV, cardiovascular. *P < 0.05. Adapted from Ref. 4.

In the ADVANCE study, 11,140 type 2 diabetics were randomly assigned to receive either standard or intensive glucose control, defined as the use of gliclazide plus any other drug required to achieve a glycosylated hemoglobin (HbA_1c_) level of 6.5% or less (Table 1).^3^ After a median follow-up of 5.0 years, the mean HbA_1c_ was lower in the intensive control group (6.5% vs. 7.3%), with a reduction in the incidence of combined major macrovascular and microvascular events primarily because of a reduction in the incidence of nephropathy. There were no significant effects of the intensive glucose control on major macrovascular events, death from cardiovascular causes, or death from any cause. Similarly, in the VADT, 1,791 suboptimally controlled type 2 diabetics, 40% with established CVD, were randomized to receive either intensive glucose control, targeting an absolute reduction of 1.5% in HbA_1c_ levels, or standard glucose control (Table 1).^5^ After a median follow-up of 5.6 years, HbA_1c_ was lower in the intensive-therapy group (6.9% vs. 8.4%). Nevertheless, there was no significant difference between the two groups in the incidence of major cardiovascular events, or in the rate of death from any cause. ACCORD was another study designed to determine whether intensive glucose control would reduce the rate of cardiovascular events (Table 1).^4^ In this study, 10,251 type 2 diabetics with median baseline HbA_1c_ of 8.1% were randomly assigned to receive either intensive therapy targeting an HbA_1c_ level within the normal range, that is, below 6.0%, or standard therapy targeting HbA_1c_ between 7.0% and 7.9%. The primary outcome was a composite of nonfatal MI, nonfatal stroke, or death from cardiovascular causes. Even though the rate of nonfatal MI was significantly lower in the intensive therapy arm, the finding of higher all-cause and cardiovascular cause mortality in this group led to discontinuation of the intensive therapy after a mean follow-up of 3.5 years (Fig. 1). Notably, hypoglycemia requiring assistance and weight gain of more than 10 kg were more frequent in the intensive therapy group. The results of the ACCORD study raised concern about not only the effectiveness but also the safety of intensive glycemic control in type 2 diabetics. Prespecified subgroup analysis of the participants in this trial suggested that patients in the intensive group without history of cardiovascular event before randomization or whose baseline HbA_1c_ level was 8.0% or less may have had fewer fatal or nonfatal cardiovascular events than did patients in the standard therapy group.

**Table 1:** Age, diabetes duration, median follow-up, HbA_1c_ values, and outcomes in the ACCORD and ADVANCE studies and the VADT

Study	Age (year)	Diabetes duration (year): intensive versus standard	Median follow-up (year)	History of CVD	HbA1c (%): intensive versus standard	Primary endpoint	Primary endpoint HR (95% CI)	All-cause mortality HR (95% CI)
ACCORD (*n* = 10,251)	62.2 ± 6.8	10 vs. 10	3.4	35%	6.4 vs. 7.5	Nonfatal MI, nonfatal stroke, or death from CVD	0.90 (0.78–1.04)	1.22 (1.01–1.46)
ADVANCE (*n* = 11,140)	66 ± 6.0	8.0 ± 6.4 vs. 7.9 ± 6.3	5.0	32%	6.53 ± 0.91 vs. 7.30 ± 1.26	Death from cardiovascular causes, nonfatal MI, or nonfatal stroke	0.94 (0.84–1.06)	0.93 (0.83–1.06)
VADT (*n* = 1,791)	60 ± 9.0	11.5 ± 8 vs. 11.5 ± 7	5.6	40%	6.9 vs. 8.4	MI, stroke, death from CVD, CHF, surgery for vascular disease, inoperable CAD, or amputation for ischemic gangrene	0.88 (0.74–1.05)	1.07 (0.81–1.42)

Several recent meta-analyses of randomized controlled trials have also investigated the effects of intensive glucose lowering on all-cause mortality, cardiovascular death, and vascular events in T2D.^6^–^10^ In the largest and most recent meta-analysis by Boussageon *et al.*, 13 studies were included.^6^ Of the 34,533 patients evaluated, 18,315 received intensive glucose-lowering treatment and 16,218 standard treatment. Intensive treatment did not significantly affect all-cause mortality or cardiovascular death. The results of this meta-analysis showed limited benefit of intensive glucose-lowering therapy on all-cause mortality and deaths from cardiovascular causes, and a 10% reduction in the risk of microalbuminuria.^6^ Results from other meta-analyses have also shown no effects of intensive glucose control on all-cause or cardiovascular mortality, while indicating a modest 15–17% reduction in the incidence of nonfatal MI in these cohorts.^7^–^10^

Several potential factors could have contributed to limit the potential benefit of intensive glucose-lowering therapies on CVD prevention in T2D individuals in studies such as the ACCORD and ADVANCE and the VADT:

1. Concomitant targeting of other potentially more potent cardiovascular risk factors, such as blood pressure and lipids, might have dampened the favorable effects of controlling hyperglycemia.
2. Intensive control of hyperglycemia could have been directed to patients unable to exhibit the expected benefit due to their specific clinical characteristics (“wrong” patients).
3. Limited benefit might have derived from using glucose-lowering drugs with no favorable impact on the global cardiovascular risk profile.
4. Glucose-lowering drugs might have produced adverse effects on the cardiovascular system by inducing weight gain and hypoglycemic events, resulting in somewhat increased risk for CVD and mortality (“imperfect” drugs).
5. Excess mortality might have potentially resulted from using too many drugs and/or too complex drug regimens, leading to undesirable drug–drug interactions with a potentially harmful impact on patients’ health.

**Figure 2:**
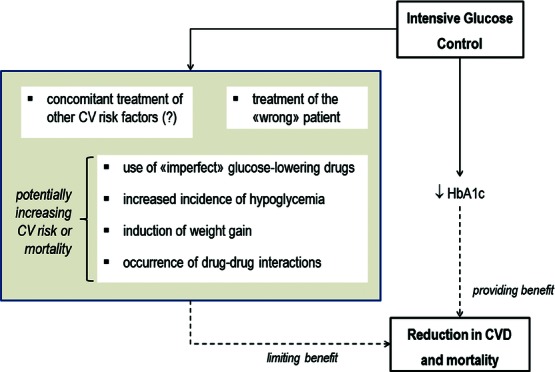
Relationship between intensive glucose control and cardiovascular outcomes and mortality in the ACCORD study and other megatrials. The potential mechanisms affecting this relationship and limiting the clinical benefit are outlined in the box on the left. CV, cardiovascular; CVD, cardiovascular disease.

These diverse factors and their potential role in the relationship between intensive glucose control and CVD/mortality are outlined in Figure 2, and will be discussed individually below.

## Limited benefit due to other therapies

Although several studies have focused on intensive glycemic control to decrease the risks of macrovascular and microvascular diseases in T2D, glucose control is only one of the factors to be considered. Comprehensive risk factor management, including blood pressure control, lipid management, weight reduction in overweight or obese individuals, and smoking cessation, are also needed. The results of the ACCORD and ADVANCE studies and the VADT should be interpreted in the context of comprehensive care of patients with diabetes. Interventions for simultaneous optimal control of comorbidities often present in type 2 diabetics, such as hypertension and hyperlipidemia, have been shown to be a more effective strategy in reducing cardiovascular risk than targeting only blood glucose levels *per se*.^11^

Evidence for an aggressive approach to lipid and blood pressure control was supported by the results from the Steno-2 study.^11^,^12^ In Steno-2, investigators used intensified multifactorial intervention with improved glycemia, renin–angiotensin system blockers, aspirin, and lipid-lowering agents and evaluated whether this approach would have an effect on the rates of death from cardiovascular causes and from any cause.^11^,^12^ The primary endpoint at 13.3 years of follow-up was the time to death from any cause. Intensive therapy was also associated with a significantly lower risk of death from cardiovascular causes and of cardiovascular events. The Steno-2 study did demonstrate a difference in levels of glycemia achieved when compared with the ACCORD study:^4^,^11^ HbA_1c_ was a mean of 8.4% at study entry and 7.9% at end of study intervention for the intensively treated group, whereas it was 8.8% at baseline and 9.0% at study end for conventional treatment. In addition, only a limited proportion of subjects in the intensively treated group reached an HbA_1c_ level of less than 6.5% (i.e., ∼15%), and this proportion was not statistically different than in the conventionally treated group, indicating poor success in achieving the prespecified glucose target and somewhat reducing the relevance of the specific intervention on the hyperglycemia component for CVD and microvascular disease prevention. The observational study has continued, and the differences observed in glycemia between intensive and conventional treatments are much less than at end of intervention. Nevertheless, over the long-term period of follow-up, intensive intervention with a varied drug regimen and lifestyle modification had sustained beneficial effects with respect to vascular complications and rates of death from any cause and from cardiovascular causes.^12^

### Blood pressure

The ACCORD study also had an embedded blood pressure trial that examined whether blood pressure lowering to systolic blood pressure (SBP) less than 120 mm Hg provided greater cardiovascular protection than a SBP of 130–140 mm Hg in T2D patients at high risk for CVD.^13^ A total of 4,733 participants were randomly assigned to intensive therapy (SBP < 120 mm Hg) or standard therapy (SBP <140 mm Hg), with the mean follow-up being 4.7 years. The blood pressure levels achieved in the intensive and standard groups were 119/64 mm Hg and 133/70 mm Hg, respectively; this difference was attained with an average of 3.4 medications per participant in the intensive group and 2.1 in the standard therapy group. The intensive antihypertensive therapy in the ACCORD blood pressure trial did not considerably reduce the primary cardiovascular outcome or the rate of death from any cause. However, the intensive arm of blood pressure control reduced the rate of total stroke and nonfatal stroke, with the estimated number needed to treat with intensive blood pressure therapy to prevent one stroke over five years being 89. There were indicators of possible harm associated with intensive blood pressure lowering (SBP < 120 mm Hg), including a rate of serious adverse events in the intensive arm. It should be noted that of the subjects investigated in the ACCORD glucose control trial ∼85% were on antihypertensive medications, and their blood pressure levels were 126.4/66.9 and 127.4/67.7 in the intensive and standard groups, respectively, a difference that was statistically significant (*P* < 0.001) (Table 2).^4^ Thus, the effects of intensive glucose lowering on the CVD and other outcomes were examined in a context in which blood pressure was being actively targeted, with possible differences between the intensively and conventionally treated cohorts.

**Table 2:** Blood pressure and lipid levels and use of statin, antihypertensive medications, and aspirin in the ACCORD and ADVANCE studies and the VADT participants at study end (adapted from Refs. 3–5)

	ACCORD (*n* = 10,251)^a^		ADVANCE (*n* = 11,140)^b^		VADT (*n* = 1,791)^c^	
			
	Standard	Intensive	Standard	Intensive	Standard	Intensive
Blood pressure (mm Hg)						
Systolic	128 ± 16	126 ± 17	137 ± 18	135 ± 17	125 ± 15	127 ± 16
Diastolic	68 ± 10	67 ± 10	74 ± 10	73 ±10	69 ± 10	68 ± 10
Cholesterol (mg/dL)						
LDL	87 ± 33	87 ± 33	103 ± 41	102 ± 38	80 ± 31	80 ± 33
HDL	49 ± 13 (♂)	49 ± 13 (♂)	48 ± 14	48 ± 14	41 ± 12	40 ± 11
	40 ± 11 (♀)	40 ± 10 (♀)				
Total	164 ± 42	163 ± 42			153 ± 40	150 ± 40
Triglycerides (mg/dL)	166 ± 114	160 ± 125	161 ± 102	151 ± 94	159 ± 104	151 ± 173
On statin (%)	88	88	48	46	83	85
On antihypertensive medications (%)	85	83	89	88	75	76
On aspirin (%)	76	76	55	57	85	86

a Intensive (target HbA_1c_ < 6%) vs. standard (HbA_1c_ 7–7.9%).

b Intensive (target HbA_1c_ < 6.5%) vs. standard (HbA_1c_ > 6.5%).

c Intensive (target HbA_1c_ 4.8–6.0%) vs. standard (HbA_1c_ 8–9.0%).

Abbreviations: HDL, high-density lipoprotein; LDL, low-density lipoprotein; ♂, men; ♀, women.

The ADVANCE study also included a blood pressure intervention trial. In this study, treatment with an angiotensin converting enzyme (ACE) inhibitor and a thiazide-type diuretic reduced the rate of death but not the composite macrovascular outcome. However, this trial had no specified targets for the randomized comparison, and the mean SBP in the intensive group (135 mm Hg) was not as low as the mean SBP in the ACCORD standard-therapy group.^14^ However, a *post hoc* analysis of blood pressure control in 6,400 patients with diabetes and coronary artery disease enrolled in the International Verapamil/Trandolapril Study (INVEST) demonstrated that tight control (<130 mm Hg) was not associated with improved cardiovascular outcomes compared with usual care (130–140 mm Hg).^15^

In the VADT, blood pressure, lipids, diet, and lifestyle were treated identically in both arms. By improving blood pressure control in an identical manner in both glucose arms, the VADT excluded the effect of blood pressure differences on cardiovascular events between treatment arms and reduced the overall risk of macrovascular complications during the trial.^5^ Participants in the VADT (*n* = 1,791) with hypertension (72.1% of total) received stepped treatment to maintain blood pressure below the target of 130/80 mm Hg in standard and intensive glycemic treatment groups. Blood pressure levels of all subjects at baseline and on-study were analyzed to detect associations with cardiovascular risk. The primary outcome was the time from randomization to the first occurrence of MI, stroke, congestive heart failure, surgery for vascular disease, inoperable coronary disease, amputation for ischemic gangrene, or cardiovascular death. From data analysis, increased risk of cardiovascular events with SBP ≥140 mm Hg emphasizes the need for treatment of systolic hypertension. Also, this study for the first time demonstrated that diastolic blood pressure (DBP) < 70 mm Hg in T2D patients was independently associated with elevated cardiovascular risk, even when SBP was on target.^16^

### Lipid profile

It has been widely demonstrated that intensive targeting of low-density lipoprotein (LDL) cholesterol contributes to CVD prevention in T2D. As a consequence, most guidelines suggest a target LDL-cholesterol level below 100 mg/dL as the primary goal in T2D individuals, with the option of achieving an LDL-cholesterol level below 70 mg/dL in those with overt CVD. Regarding the overall lipid-lowering approach in the ACCORD glucose control study, it should be observed that mean LDL cholesterol was below 90 mg/dL in both the intensive and standard glucose control arms, and that 88% of subjects were on statin therapy.^4^ Thus, the results from this study do not clarify whether lipid control and glycemic control, respectively, are related or synergistic, since the large majority of enrolled subjects were already receiving a lipid control regimen. Data from the Steno-2 trial on combined control of glucose, lipids, and blood pressure levels demonstrated significant short- and long-term benefits from this multifactorial approach.^11^ In the study, the effect seemed to be cumulative rather than synergistic.

Recent results from the ACCORD-LIPID study indicate that intensive lipid control (i.e., addition of a fibrate to statin therapy) does not reduce cardiovascular events.^17^ Specifically, the lipid-lowering arm of ACCORD failed to demonstrate any benefit of add-on therapy with fenofibrate to LDL-lowering treatment with HMG-CoA reductase inhibitors (statins) on vascular outcomes in patients with diabetes. However, data from earlier studies and from a subgroup analysis of ACCORD indicate a probable benefit of adding treatment with fibric acid derivatives to individuals with persistently elevated triglyceride levels and low high-density lipoprotein (HDL) cholesterol despite statin therapy.^17^

**Figure 3:**
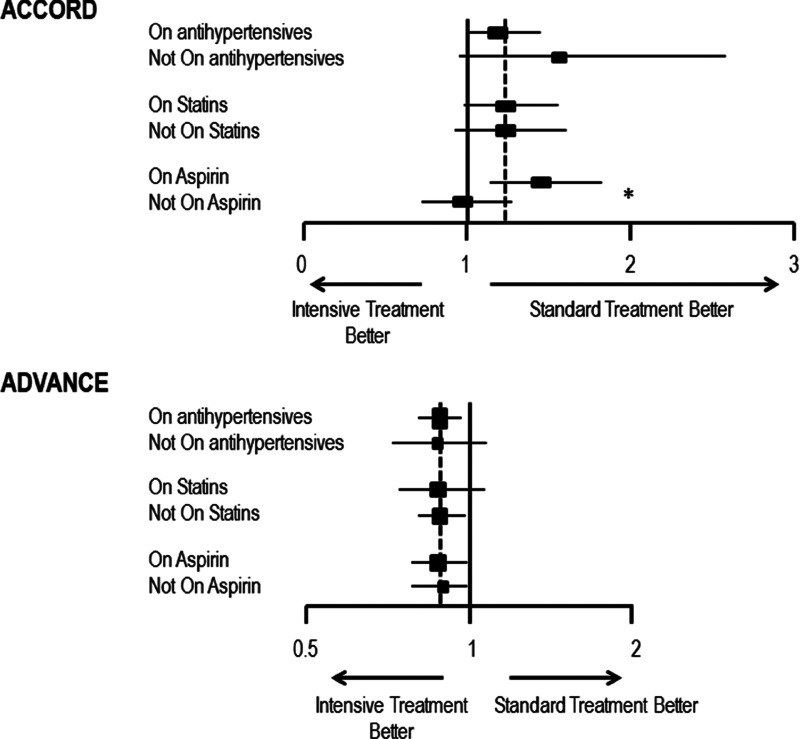
All-cause mortality in intensive versus standard glycemia groups according to use of antihypertensive medications, statins, and aspirin in the ACCORD and ADVANCE studies. *P = 0.0305 for subjects on aspirin versus subjects not on aspirin. Adapted from Refs. 3 and 18.

In the ACCORD and ADVANCE studies and the VADT, a large proportion of subjects, ranging from 55% to 85%, were also treated with aspirin (Table 2). Thus, results from ADVANCE, ACCORD, and VADT suggest that a large proportion of participants in these trials, which were being treated intensively or less intensively for glucose targets, received extensive antihypertensive, lipid-lowering, and antiplatelet medications. Median levels of SBP, SDP, and LDL cholesterol in these cohorts were also indicative of a significant proportion of them being adequately controlled for blood pressure and lipid targets (Table 2). Therefore, the possibility exists that active interventions for simultaneous control of hypertension and hyperlipidemia and use of aspirin may have affected the impact of intensive glucose control on cardiovascular outcomes in the megatrials. Subgroups analyses, however, do not apparently support this conclusion (Fig. 3). Whether patients were on antihypertensive or lipid-lowering medications was not associated with a different outcome of the intensive glucose control on mortality in ACCORD and ADVANCE patients, even though the different groups were largely unbalanced in size. Only aspirin use in the ACCORD study seemed to modulate the effects of intensive versus standard glucose control on mortality.^18^

## The “wrong patient” concept and the need for individualization of glucose targets

The ADVANCE and ACCORD studies and the VADT provide conflicting evidence of mortality risk with intensive glycemic control. These trials showed an approximate 15% reduction in nonfatal MI with no benefit or harm in all-cause or cardiovascular mortality. Potential explanations for the lack of impact of intensive glycemic control on CVD can be found in the patients’ characteristics. Indeed, these studies were of shorter duration and enrolled generally older patients than previous studies, including the DCCT and UKPDS in which the intensive control had shown better outcomes. In addition, mean diabetes duration was longer and a greater portion of patients had established CV disease in the megatrials (approximately 32–40%) than in earlier trials (Table 1). It is also possible that the follow-up of these studies was too short to detect a clinical benefit. Consistent with this hypothesis, in the UKPDS no macrovascular benefit was noted in the intensive control arm in the first 10 years of follow-up. Nevertheless, posttrial monitoring for an additional 10 years (UKPDS 80) revealed a 15% risk reduction in MI and 13% reduction in all-cause mortality in the intensive treatment group.^19^

A possible explanation is that the wrong patients were investigated in the megatrials (Fig. 2). Indeed, the population of the ACCORD study may not represent the average patient with T2D in clinical practice. Participants in this trial had T2D on average for 10 years at the time of enrollment, had higher HbA_1c_ levels than most type 2 diabetic patients in the United States and most Western countries today (average of 8.2% at baseline), and had known heart disease or at least two risk factors in addition to diabetes, such as high blood pressure, high cholesterol levels, obesity, and smoking.^4^ In the UKPDS, the benefits of intensive glycemic control on CVD were observed only after a long duration of intervention in newly diagnosed younger patients.^2^ In older patients with T2D with longer disease duration, atherosclerotic disease may already have been established and thus intensive glucose control may have had little benefit. Conversely, patients with shorter disease duration, lower HbA_1c_, and/or lack of established CVD might have benefited significantly from more intensive glycemic control.^20^,^21^ Relevant to this concept, the VADT showed that advanced CVD, as demonstrated by computed tomography (CT)-detectable coronary artery calcium, was associated with negative outcomes. In a substudy cohort of 301 T2D participants in VADT, the ability of intensive glucose therapy compared with standard therapy to reduce cardiovascular events was examined based on the extent of coronary atherosclerosis as measured by a CT-detectable coronary artery calcium score (CAC). The data showed that there was a progressive diminution of the benefit of intensive glucose control with increasing CAC. In patients with CAC ≤ 100, 1 of 52 individuals experienced an event (HR for intensive therapy = 0.08; range, 0.008–0.770; *P* = 0.03), whereas 11 of 62 patients with a CAC > 100 had an event (HR = 0.74; range, 0.46–1.20; *P* = 0.21). Thus, this subgroup analysis indicates that intensive glycemic therapy may be most effective in those with less extensive coronary atherosclerosis.^22^

Why does intensive treatment of hyperglycemia appear to be ineffective in reducing cardiovascular events in T2D with advanced atherosclerosis? Two potential mechanisms may be involved. First, once the atherosclerotic plaque has developed, modified lipoproteins, activated vascular cells, and altered immune cell signaling may generate a self-propagating process that maintains atherogenesis, even in the face of improved glucose control. Second, advanced glycosylation end-product formation, which may be involved in CVD, is not readily reversible and may require more than three to five years of intensive glucose control to be reverted. Thus, the presence of multiple cardiovascular risk factors or established CVD may have reduced the benefits of intensive glycemic control in the high-risk cohort of ACCORD, ADVANCE, and VADT compared with the low-risk population of the UKPDS cohort, of whom only a minority had prior CVD.^23^ The presence of long-standing disease and prolonged prior poor glycemic control may be additional factors accelerating the progression of atherosclerotic lesions in T2D.

The goal of individualizing HbA_1c_ targets has gained more attraction after these recent clinical trials in older patients with established T2D failed to show a benefit from intensive glucose-lowering therapies on CVD outcomes. Recommendations suggest that the goals should be individualized, such that (1) certain populations (children, pregnant women, and elderly patients) require special considerations and (2) more stringent glycemic goals (i.e., a normal HbA_1c_ < 6.0%) may further reduce complications at the cost of increased risk of hypoglycemia.^24^ For the latter, the recommendations also suggest that less intensive glycemic goals may be indicated in patients with severe or frequent hypoglycemia. With regard to the less intensive glycemic goals, perhaps consideration should be given to the high-risk patient with multiple risk factors and CVD, as evaluated in the ACCORD study.^4^ Thus, aiming for a HbA_1c_ of 7.0–8.0% may be a reasonable goal in patients with very long duration of diabetes, history of severe hypoglycemia, advanced atherosclerosis, significant comorbidities, and advanced age/frailty (Table 3), even though with what priority each one of these criteria should be considered is not clear at present (current recommendations from scientific societies also do not provide this specific information). In younger patients without documented macrovascular disease or the above-mentioned conditions, the goal of attaining an HbA_1c_ < 6.5–7.0% may provide long-lasting benefits. In patients with limited life expectancy, more liberal HbA_1c_ values may be pursued. In determining the HbA_1c_ target for CVD prevention, one should consider that at least three to five years are usually required before possible differences in the incidence of nonfatal MI in T2D are observed.^2^ Thus, a clinical setting that allows tight glucose control to be implemented for this period of time should be available. Finally, an excess mortality was observed in the ACCORD study in those T2D individuals who showed an unexpected increase in HbA_1c_ levels upon institution of intensive glucose control.^25^ Accordingly, patients exhibiting the pattern of worsening glycemic control when exposed to intensive treatment should be set at higher glucose targets (Table 3).

**Table 3:** Potential criteria for individualization of glucose targets in type 2 diabetes^1^–^3^,^5^,^25^,^29^

Criterion	HbA_1c_ < 6.5–7.0%	HbA_1c_ 7.0–8.0%
Age (years)	<55	>60
Diabetes duration (years)	<10	>10
Life expectancy (years)	>5	<5
Possibility to perform IGC for >5 years	Yes	No
Usual HbA_1c_ level (%)	<8.0	>8.0
CVD	No	Yes
Prone to hypoglycemia	No	No
Reduction of HbA_1c_ level upon IGC	Yes	No

Abbreviations: CVD, cardiovascular disease; IGC, intensive glucose control.

The above guidelines are apparently incorporated into the updated version of the American Diabetes Association and the European Association for the Study of Diabetes recommendations on the management of hyperglycemia in nonpregnant adults with T2D. The new recommendations are less prescriptive and more patient centered. Individualized treatment is explicitly defined as the cornerstone of success. Treatment strategies should be tailored to individual patient needs, preferences, and tolerances and based on differences in age and disease course. Other factors affecting individualized treatment plans include specific symptoms, comorbid conditions, weight, race/ethnicity, sex, and lifestyle.^26^

## Current “imperfect” glucose-lowering drugs

The inability of ACCORD, ADVANCE, and VADT to demonstrate significant reductions in CVD outcomes with intensive glycemic control also suggests that current pharmacological tools for treating hyperglycemia in patients with more advanced T2D may have counterbalancing consequences for the cardiovascular system. The available agents used to treat diabetes have not been conclusively shown to reduce macrovascular disease and, in some instances, their chronic use may promote negative cardiovascular effects in diabetic subjects despite improvement of hyperglycemia. Importantly, these adverse cardiovascular side effects appear in several instances to be directly due to the mode of drug action. Selection of a treatment regimen for patients with T2D includes evaluation of the effects of medications on overall cardiovascular risk.^27^

Sulfonylureas have the benefit of acting rapidly to lower glucose levels but, unfortunately, on a long-term basis these drugs do not preserve β cell function, and after the first year of therapy glucose levels start to rise progressively. There are a number of controversial studies suggesting that sulfonylureas may increase the risk for CVD in patients with diabetes. Although this is a topic that remains to be settled, it appears to be clear that these drugs do not directly reduce the risk for CVD.^19^,^28^,^29^

Thiazolidinediones may cause weight gain, and this effect is often associated with blood volume expansion. In addition, thiazolidinediones have been associated with an increased risk of congestive heart failure and, in the case of rosiglitazone, an increased risk of CVD.^30^,^31^ Currently, rosiglitazone has been discontinued in the European market and its use in the United States has been largely restricted. By contrast, a meta-analysis of trials of the thiazolidinedione pioglitazone indicated the possibility of an ischemic cardiovascular benefit.^32^ Pioglitazone has been shown to exert a number of beneficial effects on cardiovascular risk factors: it lowers blood sugar levels, improves diabetic dyslipidemia, and is effective in raising HDL cholesterol and lowering triglycerides and apolipoprotein B100, while displaying a neutral effect on LDL cholesterol. Pioglitazone also reduces the levels of C-reactive protein and a number of inflammatory markers that are increased in T2D, while increasing adiponectin, which adds to its insulin-sensitizing and anti-atherogenic potential.^33^–^35^ However, in spite of the ample preclinical and clinical evidence based on intermediate endpoints, the results from large randomized controlled studies with clear clinical endpoints have not fully met expectations. In the PROactive (PROspective pioglitAzone Clinical Trial In macroVascular Events) study, pioglitazone did not significantly reduce the primary composite cardiovascular endpoint (only 10% reduction, which was nonsignificant).^36^ Nevertheless, the major cardiovascular events (MACE) endpoint of cardiovascular death, stroke, and MI was modestly but significantly decreased by 16% (*P* = 0.027), and several meta-analyses of all of the published pioglitazone data, including an analysis by the FDA, have supported this magnitude of reduction.^37^,^38^ In a *post hoc* subgroup analysis on patients with previous MI, pioglitazone reduced fatal and nonfatal MI by 28% and acute coronary syndrome by 37%.^39^

It has been suggested that insulin, particularly in high doses, may be atherogenic.^40^ All of the long-term insulin trials also have shown that insulin therapy is associated with weight gain and, of course, weight gain and obesity are independent risk factors for CVD.^41^ Some studies have also shown that insulin stimulates *de novo* lipogenesis and, in some cases, may aggravate underlying dyslipidemia.^42^ Thus, insulin may not be the ideal drug if one is concerned about reducing cardiovascular risk in T2D associated with overweight or obesity.

Glucagon-like peptide-1 (GLP-1)–based therapies may exert potential favorable effects on the CV system, in addition to targeting of hyperglycemia in T2D individuals. It should be noted that the use of GLP-1 receptor agonists and DPP-1 inhibitors in ACCORD, ADVANCE, and VADT has been very limited or lacking. GLP-1 receptor agonists have a positive impact on cardiovascular risk factors that are not specifically addressed by standard diabetes medications (Table 4).^43^,^44^ Both liraglutide and exenatide reduce excess weight and blood pressure and improve the lipid profile. Further, GLP-1 analogues have been documented to have a direct natriuretic effect and a direct action of endothelial vasodilatation.^45^,^46^ Additional data show that liraglutide reduces several markers of cardiovascular risk, such as C-reactive protein, type 2 natriuretic peptide, and PAI-1.^47^ A potential role of these drugs in the context of heart failure or coronary artery disease is also suggested by the evidence that continuous infusion of GLP-1 is associated with improvements in left ventricular function in patients with acute MI and severe systolic dysfunction and in patients with congestive heart failure.^48^,^49^ The protective effect of GLP-1 and GLP-1 analogues on ischemic and reperfusion injury, largely mediated by inhibition of apoptosis, have been documented in several animal models, showing that these compounds may reduce infarct size and improve outcomes after experimental MI.^50^–^52^ A potent antiapoptotic effect on human cardiac progenitors has recently been reported by a recent study.^53^ In the light of these findings and the ability of GLP-1 receptor agonists to reduce multiple cardiovascular risk factors and to exhibit potential favorable effects on the cardiovascular system, it has been postulated that these drugs may reduce cardiovascular events and mortality; however, direct evidence for such effects is lacking at present. Long-term studies are underway with liraglutide, the long-acting preparation of exenatide, exenatide LAR, and the GLP-1 analogues lixisenatide and dulaglutide to assess whether in large patient cohorts (in excess of 5,000) followed for five years or longer incretin-based medications can reduce the incidence of CVD.^54^

**Table 4:** Potential cardiovascular impact of incretin-based therapies

Effects (direct and indirect)
Myocardial protection against ischemia
Changes in CV risk factors (traditional, nontraditional)
Arterial vasodilation
Mechanisms (cellular and biochemical)
Inhibition of apoptosis (endothelial cells, myocardiocytes, and cardiac progenitors)
Antagonism of cytokines → inhibition of inflammation and atherogenesis
eNOS activation and endothelial-dependent vasodilation
DPP-4 dependent (GLP-1 independent?) pathways
Information from clinical studies
Reduced incidence of MACE (*post hoc* analyses from phase II/III trials)
Need for long-term intervention trials

Abbreviations: CV, cardiovascular; DPP-4, dipeptidylpeptidase-4; eNOS, endothelial nitric oxide synthase; GLP-1, glucagon-like peptide-1; MACE, major adverse cardiovascular events.

Dipeptidyl peptidase-4 (DPP-4) inhibitors do not reduce body weight but are certainly weight neutral. From the standpoint of weight neutrality, this is an advantage of the DPP-4 inhibitor drug class, since other glucose-lowering drugs, such as thiazolidinediones, sulfonylureas, and insulin, tend to promote weight gain. Although evidence is limited, DPP-4 inhibitors have also been shown to produce some beneficial effects on blood pressure and lipid levels. For example, in patients with moderate hypertension and no diabetes, the DPP-4 inhibitor sitagliptin (50–100 mg, OD) reduced systolic and diastolic blood pressure by more than 2 mm Hg compared with placebo.^55^ Sitagliptin also reduced plasma triglyceride levels by 10–15% and increased HDL cholesterol by more than 5% in doses of 25–100 mg daily as monotherapy in patients with T2D over 12 weeks.^56^ Moreover, DPP-4 inhibitors may exploit the favorable actions of GLP-1 on the cardiovascular system by increasing the bioavailability of this hormone and, additionally, may potentially exert cardioprotective effects via non GLP-1–mediated mechanisms involving other peptides.^54^ Currently, the effects of the DPP-4 inhibitors on cardiovascular outcomes are being addressed in long-term intervention studies using sitagliptin, saxagliptin, and linagliptin.^54^

## Role of hypoglycemia and weight gain in response to treatment intensification

Excess mortality with intensive glycemic treatment in the recent megatrials could be potentially related to the negative effects of hypoglycemic events. However, it is unclear whether this was casual or related to hypoglycemic events identifying fragile patients.

Hypoglycemia was increased in all the intensive therapy groups of the three megatrials. Rates of serious hypoglycemia were highest in the ACCORD study and the VADT.^3^–^5^ Severe hypoglycemia has been suggested to be responsible for excess deaths in the ACCORD study; the rate of hypoglycemic events requiring medical assistance was 10.5% in the intensive treatment group compared to 3.5% in the conventional therapy group.^4^ In the VADT, 21.2% of patients in the intensive treatment group had at least one serious adverse event related to hypoglycemia compared with 9.9% in the conventional therapy group.^5^ In the ADVANCE study, 2.7% of the intensive group compared with 1.5% of the conventional therapy group had a severe hypoglycemic event.^3^ In the UKPDS, the insulin treatment group had the most episodes of serious hypoglycemia (mean proportion 2.3% per year over 10 years) followed by the sulfonylurea group (0.4–0.6% per year).^41^ It is biologically plausible that severe hypoglycemia could increase the risk of cardiovascular death in participants with high underlying CVD risk. This might be further confounded by the development of hypoglycemia unawareness, particularly in patients with coexisting cardiovascular autonomic neuropathy (a strong risk factor for sudden death). Death resulting from a hypoglycemic event may be mistakenly ascribed to coronary artery disease, because there may not have been a blood glucose measurement and because there are no anatomic features of hypoglycemia detected postmortem.^57^

In healthy individuals, acute hypoglycemia provokes sympathoadrenal activation and counter-regulatory hormonal secretion.^58^ This mechanism plays an important role in protecting the brain from neuroglycopenia through altering blood flow in the brain and other metabolic changes to restore blood glucose to normal. In healthy individuals, this does not cause detrimental effects. In patients with diabetes who have already developed endothelial dysfunction, acute hypoglycemia leads to acute hemodynamic and hematologic changes, which ultimately lead to increased risk of tissue ischemia and major vascular events, including MI and stroke.^57^ Indeed, hypoglycemia stimulates the release of catecholamines, which increase myocardial contractility, workload, and cardiac output, potentially inducing myocardial ischemia in patients with coronary heart disease. Hypoglycemia is also associated with a significant lengthening of the corrected QT interval in subjects with diabetes, predisposing to a high risk of ventricular tachycardia and sudden death.^59^ Increased catecholamine secretion and hyperinsulinemia may lead to hypokalemia during hypoglycemic events, thereby potentiating cardiac repolarization abnormalities. Several inflammatory markers, including C-reactive protein, interleukin (IL)-6, IL-8, tumor necrosis factor alpha, and endothelin-1, have been shown to be increased during hypoglycemia, thus potentiating endothelial injury and abnormalities in coagulation and platelet function and resulting in increased risk for cardiovascular events.^57^ Recent studies suggest that vessel wall stiffness is increased during hypoglycemia in patients with type 1 diabetes (T1D) of longer duration than those with shorter duration.^60^ Thus, hypoglycemia may directly contribute to the increased risk of cardiovascular events and mortality, especially in patients with longer disease duration.

However, recent *post hoc* analyses and follow-up data from the ACCORD study have somehow dampened this concept; severe hypoglycemia was associated with an increased risk of death within each study arm, but among participants who experienced at least one episode of hypoglycemia the risk of death was higher in the standard arm than in the intensive arm (4.5% vs. 2.8%, respectively, annualized mortality rates for hypoglycemic events requiring medical assistance, *P* = 0.009).^61^ Furthermore, a greater drop in HbA_1c_ levels within the first four months of the study, occurring particularly in the intensive arm, was not associated with an increased risk for hypoglycemia.^61^ Also, the excess deaths from any cause (primarily cardiovascular), observed in the intensive arm at the end of the intervention for 3.7 years, persisted after a follow-up period of 1.3 years, in spite of similar HbA_1c_ levels, use of glucose-lowering medications, and rates of severe hypoglycemia in the intensive and standard intervention groups.^62^ Together, these analyses have suggested that hypoglycemia may not account for the excess mortality associated with intensive glucose lowering in the ACCORD study and may instead be a marker of vulnerability that increases the risk for death in patients with diabetes, even when glycemia is controlled less intensively.^61^ A similar conclusion was drawn from analyses of data in the ADVANCE study.^63^ On the basis of these observations, indiscriminate application of intensive glucose-lowering therapies that can provoke dangerous hypoglycemia in elderly people with T2D or in patients with overt CVD should be avoided.

Another plausible mechanism for increased mortality in the ACCORD study includes weight gain. The ACCORD study and the VADT enrolled overweight and obese patients, with mean BMI values of 32 kg/m^2^ in ACCORD and of 31 kg/m^2^ in VADT.^4^,^5^ The ADVANCE trial started with fewer overweight patients with an average BMI of 28 kg/m^2^.^3^ Patients in both trials had the greatest weight gain in the intervention groups: the ACCORD intensive group gained 3.5 kg compared with 0.4 kg in the standard group; the VADT intensive group gained 8.2 kg compared to 4.1 kg in the standard group. The UKPDS also had significant weight gain in the insulin and sulfonylurea arms (insulin, 4.0 kg; glybenclamide, 1.7 kg; chlorpropamide, 2.6 kg).^2^ There was much less difference in weight gain between groups in the ADVANCE and the UKPDS on metformin.^2^,^64^

## Drug–drug interactions

The use of multiple combinations of glucose-lowering medications was required in the intensive arms of the megatrials in order to achieve near-normal HbA_1c_ levels. For example, in the ACCORD study, 42% of participants in the intensive therapy group received three or more classes of oral agents, either alone (17%) or in combination with insulin (25%), whereas such combinations were used in 19% of the participants in the standard therapy group.^4^ It is likely that the increase in mortality in the ACCORD study was related to the overall treatment strategies for intensifying glycemic control in the study population. Therefore, another possible cause of the higher death rate in the intensive treatment group of the ACCORD study, compared with the ADVANCE study and the VADT, includes undetected adverse interactions among the various drug classes used at high doses (Fig. 2). Whether similar findings would have been observed with newer glucose-lowering therapies or different drug combinations is unknown.

## Conclusions

The recent publication of the findings from the ACCORD trial, the ADVANCE trial, and the VADT has provided important insights into the balance of risks and benefits associated with the use of more intensive glucose control in patients with T2D. However, the relationship between control of hyperglycemia and cardiovascular risk remains largely controversial.

The results of the ACCORD study indicate for the first time that aggressive treatment to normalize blood glucose levels may not be appropriate in certain high-risk populations, possibly due to an increase in the risk of hypoglycemia and weight gain. Thus, individualization of therapy becomes important. The HbA_1c_ goal for T2D patients should be individualized based on the duration of diabetes, preexisting CVD, hypoglycemia unawareness, comorbidities, response to therapy, and frailty (Table 3). For selected individual patients, lower HbA_1c_ goals than the general goal of <7.0% should be considered if this can be achieved without significant hypoglycemia or other adverse effects of treatment. Such patients might include those with short duration of diabetes, long life expectancy, and no significant CVD. Conversely, less stringent HbA_1c_ goals than the general goal of <7% may be appropriate for patients with a history of severe hypoglycemia, limited life expectancy, advanced microvascular or macrovascular complications, extensive comorbid conditions (Table 3), or those with long-standing diabetes in whom the general goal is difficult to be attained despite diabetes self-management education, appropriate glucose monitoring, and effective doses of multiple glucose-lowering agents including insulin. Also, cardiovascular risk reduction requires comprehensive assessment and management of all known contributing risk factors (Table 5). Smoking cessation and lipid, blood pressure and weight control should be emphasized.

**Table 5:** Multifactorial intervention for CVD prevention in T2D

	“The lower the better”	More aggressive therapy beneficial in specific patients	Specific drug regimens with prominent CV benefit
HbA_1c_	No^2^,^4^,^5^	Yes^2^,^4^,^5^ (young/long life expectancy, short duration of T2D, well-controlled T2D, no CVD, no hypoglycemia, HbA_1c_ reduction upon IGC)	Yes (?)^36^,^39^,^64^ (metformin, pioglitazone)
Blood pressure	No^13^	Usually No^24^ (except when proteinuria >1 g/day)	No evidence
Lipids	Yes^24^	Yes^24^ (very high CV risk; evidence of CVD)	Yes (?)^17^ (Fenofibrate plus statin in high trygliceride/low HDL-C phenotype)

Abbreviations: CV, cardiovascular; CVD, cardiovascular disease; HDL-C, high-density lipoprotein cholesterol; IGC, intensive glucose control; T2D, type 2 diabetes; ?, evidence derived from a single intervention trial.

The advent in clinical practice of new classes of drugs, such as the incretin-based therapies, could be useful to this end. Although only limited clinical data are currently available on the utility of such therapies, they seem to meet the ideal profile a glucose-lowering drug that has long-term efficacy, low risk of hypoglycemia, cardiovascular protection, neutral effect on body weight or weight loss, long-term safety, and tolerability. At present, GLP-1 receptor agonists and DPP-4 inhibitors seem promising. If data on cardiovascular efficacy of these drugs is borne out by the long-term ongoing studies, they could acquire a prominent role among therapeutic options, not only as add-on treatments, in cases of inadequate glucose control with metformin, but also as an early strategy to reduce the burden of diabetes and its vascular complications.
